# Comparison of anti-inflammatory effects and high-density lipoprotein cholesterol levels between therapy with quadruple-dose rosuvastatin and rosuvastatin combined with ezetimibe

**DOI:** 10.1186/1476-511X-12-9

**Published:** 2013-02-04

**Authors:** Daisuke Yamazaki, Masaru Ishida, Hiroyuki Watanabe, Kiyoshi Nobori, Yasunori Oguma, Yutaka Terata, Takashi Koyama, Kenji Iino, Toshimitsu Kosaka, Hiroshi Ito

**Affiliations:** 1 Department of Cardiovascular Medicine, Akita University Graduate School of Medicine, Hondo 1-1-1, Akita, 010-8543, Japan

**Keywords:** Statin, Secondary prevention, Coronary artery disease

## Abstract

**Background:**

Statins are frequently administered to reduce low-density lipoprotein cholesterol (LDL-C) and vascular inflammation, because LDL-C and high sensitive C-reactive protein (hs-CRP) are associated with high risk for cardiovascular events. When statins do not reduce LDL-C to desired levels in high-risk patients with coronary artery disease (CAD), ezetimibe can be added or the statin dose can be increased. However, which strategy is more effective for treating patients with CAD has not been established. The present study compares anti-inflammatory effects and lipid profiles in patients with CAD and similar LDL-C levels who were treated by increasing the statin dose or by adding ezetimibe to the original rosuvastatin dose to determine the optimal treatment for such patients.

**Methods:**

46 patients with high-risk CAD and LDL-C and hs-CRP levels of >70 mg/dL and >1.0 mg/L, respectively, that were not improved by 4 weeks of rosuvastatin (2.5 mg/day) were randomly assigned to receive 10 mg (R10, n = 24) of rosuvastatin or 2.5 mg/day of rosuvastatin combined with 10 mg/day of ezetimibe (R2.5/E10, n = 22) for 12 weeks. The primary endpoint was a change in hs-CRP.

**Results:**

Baseline characteristics did not significantly differ between the groups. At 12 weeks, LDL-C and inflammatory markers (hs-CRP, interleukin-6, tumour necrosis factor-alpha and pentraxin 3) also did not significantly differ between the two groups (LDL-C: R10 vs. R2.5/E10: -19.4 ± 14.2 vs. -22.4 ± 14.3 mg/dL). However, high-density lipoprotein cholesterol (HDL-C) was significantly improved in the R10, compared with R2.5/E10 group (4.6 ± 5.9 vs. 0.0 ± 6.7 mg/dL; p < 0.05).

**Conclusion:**

Both enhanced therapies exerted similar anti-inflammatory effects under an equal LDL-C reduction in patients with high-risk CAD despite 2.5 mg/day of rosuvastatin. However, R10 elevated HDL-C more effectively than R2.5/E10.

**Trial registration:**

UMIN000003746

## Background

The relationship between coronary artery disease (CAD) and serum levels of low-density lipoprotein cholesterol (LDL-C) has been documented in detail [1-3]. Several large trials have shown that statins (3-hydroxymethylglutaryl coenzyme A reductase inhibitors) can reduce LDL-C and thus improve clinical outcomes after atherosclerotic cardiovascular events [4,5].

Inflammation is also closely associated with CAD outcomes. Serum concentrations of high-sensitive C-reactive protein (hs-CRP), which is the most common inflammatory biomarker, can also be used to predict the risk of future myocardial infarction [6,7]. Statins can reduce hs-CRP [8-12] and exert pleiotropic anti-inflammatory effects that might be unrelated to lowering cholesterol. As a result, statins are commonly applied as secondary prevention for high-risk patients with high LDL-C and hs-CRP levels, although no guidelines have established a target for hs-CRP.

On the other hand, cholesterol absorption in hypercholesterolemic patients is often blocked using statins in combination with ezetimibe, which is a cholesterol transporter Niemann-Pick C1-Like 1 protein inhibitor. Several investigators have reported that ezetimibe also has both anti-inflammatory [13] and pleiotropic effects [14]. However, only a few studies have specifically examined the effects of ezetimibe, whereas statins have been investigated in detail.

The guidelines of the European Society of Cardiology and the European Atherosclerosis Society (ESC/EAS) [15], and of the American Diabetes Association and the American College of Cardiology (ADA/ACC) [16] recommend that LDL-C should be lowered to < 70 mg/dL in high-risk patients with CAD. When statins cannot achieve this level in such patients, alternative strategies usually comprise increasing the dose of statins or adding ezetimibe to the original statin dose. Although routinely applied, which of these strategies is the most effective for high-risk patients with CAD has not been determined. The effects of escalating the dose of rosuvastatin and of adding ezetimibe under equal LDL-C levels have not been compared.

The present study compares anti-inflammatory effects and lipid profiles after increasing the dose of rosuvastatin or adding ezetimibe to determine the optimal strategy for treating patients with CAD and similar LDL-C levels.

## Methods

### Subjects

All enrolled male and female patients were aged a median of 73 (range, 42–84) years and had undergone percutaneous coronary intervention for CAD. All of them had LDL-C levels above the target for secondary prevention in patients with high-risk CAD (>70 mg/dL) despite treatment with 2.5 mg/day of rosuvastatin for 4 weeks. All of them also had hs-CRP levels >1.0 mg/L, which is the cut-off value for a high risk of future CAD demonstrated in the Hisayama study [17].

Patients were excluded if they had a history of statin-induced myopathy, hypersensitivity reactions to statins or ezetimibe, acute myocardial infarction within the past month, acute or chronic inflammatory disease (hs-CRP > 10 mg/L, body temperature > 37°C), acute hepatitis, acute exacerbation of chronic hepatitis, liver cirrhosis, liver cancer, jaundice, chronic renal failure (creatinine ≥ 2.0 mg/dL or an estimated glomerular filtration rate < 30 mL/min/1.73 m^2^).

### Study design

**Figure 1:**
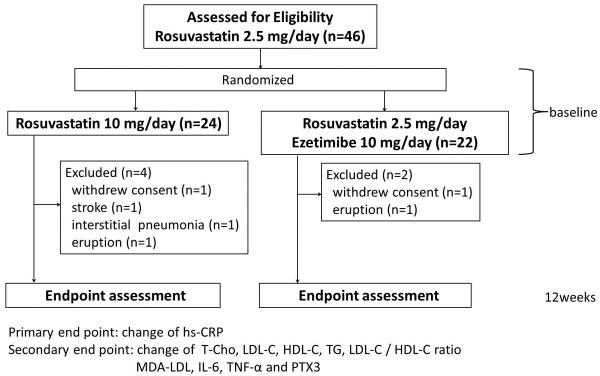
**Study flow.** HDL-C, high-density lipoprotein cholesterol; hs-CRP, high sensitivity-C-reactive protein; IL-6, interleukin-6; LDL-C, low-density lipoprotein cholesterol; MDA-LDL, malondialdehyde-modified-low-density lipoprotein; T-Cho, total cholesterol; TG, triglycerides; TNF-α, tumour necrosis factor-α; PTX3, pentraxin 3.

This prospective, open-label, randomized, parallel group study proceeded at Akita University Hospital, Yuri General Hospital and Yamamoto General Hospital and is registered with UMIN under the number UMIN000003746. Figure 1 shows the study protocol.

In accordance with previous findings [18-20], a quadruple dose of statin and the normal dose of statin combined with ezetimibe were considered to equally decrease LDL-C levels. Based on this assumption, we compared the effects of rosuvastatin (10 mg/day; R10) with those of rosuvastatin (2.5 mg/day) plus ezetimibe (10 mg/day; (R2.5/E10) for 12 weeks.

Patients were randomly assigned using a centralised allocation at four weeks after enrolment to groups that would receive either R10 or R2.5/E10. All patients provided written informed consent to participate in the study. The first patient was enrolled in July 2010 and the last completed the study in June 2012. The Akita University Hospital Ethics Committee approved the study protocol, which proceeded according to the Declaration of Helsinki.

### End points

The primary endpoint was a change in hs-CRP from baseline after 12 weeks in each group. The secondary endpoints were changes in levels of total cholesterol (T-Cho), LDL-C, high-density lipoprotein cholesterol (HDL-C), triglycerides (TG), LDL-C/HDL-C ratio, malondialdehyde-modified low-density lipoprotein cholesterol (MDA-LDL), interleukin-6 (IL-6), tumour necrosis factor-α (TNF-α) and pentraxin 3 (PTX3) at 12 weeks after randomization.

### Laboratory assessments

Fasting blood samples were collected at baseline and at 4, 8 and 12 weeks thereafter. T-Cho, TG and HDL-C were measured enzymatically using an autoanalyzer (Hitachi Co., Tokyo, Japan). We estimated LDL-C using the Friedewald formula (LDL-C = TC - HDL-C - TG × 0.2) except if patients had current TG levels of >400 mg/dL. However, none of our patients had TG > 400 mg/dL. Serum hs-CRP was measured by nephelometry, IL-6 was measured using a chemiluminescent enzyme immunoassay and MDA-LDL, TNF-α and PTX3 were measured using enzyme-linked immunosorbent assays.

### Statistical analyses

This exploratory study assembled data to verify the favourable pleiotropic effects of statin compared with ezetimibe. Previous studies [21,22] have determined that a sample of 126 patients would enable a power of 80%, with a two-tailed type1 error of 0.05, to detect a difference of 1.0 mg/L between the geometric means of hs-CRP in two groups assuming a standard deviation of 2.0 mg/L. However, to collect this many patients with hs-CRP > 1.0 mg/L who were administered with rosuvastatin 2.5 mg was difficult. Therefore, we redesigned this study as an exploratory effort to assemble the data required to verify the above and determined the sample size considering the operability of this study.

All data were statistically analysed using GraphPad Prism version 5 (GraphPad Software, San Diego, CA, USA) and are presented as means ± standard deviation, or as medians (25^th^ and 75^th^ percentiles) when the distribution was not normal. Lipid profiles as well as hs-CRP, IL-6, TNF-α and PTX3 levels at 4, 8 and 12 weeks were compared with baseline values using Student’s *t*-test. Changes in the lipid profile and inflammatory markers were compared between the two groups using an analysis of variance (ANOVA). Differences were considered statistically significant at p < 0.05.

## Results

Among 46 patients who initially enrolled in the present study, 24 and 22 received R10 and 22 R2.5/E10, respectively. Four patients in the R10 group withdrew from treatment having decided not to complete the study, eruption, stroke and interstitial pneumonia (n = 1 each) and two in the R2.5/E10 group withdrew having decided not to complete the study and eruption (n = 1 each).

Table 1 summarizes the baseline characteristics of the two groups, which did not significantly differ with respect to age, body mass index, sex, prevalence of coronary risk factors, or medication at the time of randomization, lipid profiles and inflammatory markers.

**Table 1:** Patient characteristics at randomization

	**Rosuvastatin 10 mg/day**	**Rosuvastatin 2.5 mg/day Ezetimibe 10 mg/day**	**P**
	**(n = 24)**	**(n = 22)**	
Age (y)	71.8 ± 8.2	70.1 ± 9.6	0.54
Body mass index (kg/m2)	26.0 ± 2.8	24.4 ± 3.2	0.08
Male, n (%)	15 (62.5)	14 (63.6)	0.94
Current or former smoker, n (%)	15 (62.5)	11 (50.0)	0.73
Hypertension, n (%)	19 (79.0)	17 (77.0)	0.88
Diabetes mellitus, n (%)	10 (41.7)	8 (36.4)	0.71
History of MI, n (%)	13 (54.2)	11 (50.0)	0.53
Medication			
Beta-blockers, n (%)	11 (45.8)	10 (45.5)	1.00
ACEIs or ARBs	19 (79.2)	17 (77.3)	0.88
Calcium channel blockers, n (%)	15 (62.5)	8 (36.4)	0.08
Oral hypoglycaemics, n (%)	9 (37.5)	8 (36.4)	0.94
Insulin, n (%)	4 (16.7)	1 (4.5)	0.19
Aspirin, n (%)	24 (100)	22 (100)	1.00
Clopidogrel, n (%)	18 (75.0)	13 (59.1)	0.25
Laboratory data			
T-Cho (mg/dL)	168.0 ± 17.4	164.0 ± 23.3	0.51
LDL-C (mg/dL)	88.5 ± 12.9	84.3 ± 14.5	0.30
HDL-C (mg/dL)	46.4 ± 11.6	49.9 ± 12.2	0.33
TG (mg/dL)	165.4 ± 78.9	149.4 ± 103.9	0.56
LDL-C/HDL-C ratio	1.96 ± 0.50	1.77 ± 0.41	0.18
MDA-LDL (U/L)	104.6 ± 26.8	94.2 ± 18.8	0.14
hs-CRP (mg/L)	2.0 ± 2.0	2.5 ± 2.5	0.32
IL-6 (pg/mL)	9.4 ± 25.9	5.4 ± 6.8	0.37
TNF-α (pg/mL)	4.4 ± 8.9	7.6 ± 13.9	0.37
PTX3 (ng/mL)	1.95 ± 1.23	2.02 ± 0.79	0.30

Data are shown as means ± standard deviation. ACEI, angiotensin-converting enzyme inhibitor; ARB, angiotensin receptor blocker; HDL-C, high-density lipoprotein cholesterol; hs-CRP, high sensitivity C-reactive protein; IL-6, interleukin 6; LDL-C, low-density lipoprotein cholesterol; MDA-LDL, malondialdehyde-modified-low-density lipoprotein; MI, myocardial infarction; T-Cho, total cholesterol; TG, triglycerides; TNF-α, tumour necrosis factor-α; PTX3, pentraxin 3.

**Table 2:** Lipid profiles at baseline, 4, 8 and 12 weeks after randomization

	**Rosuvastatin 10 mg/day**				
	**Baseline**	**4**	**8**	**12**	**12 weeks - baseline**
T-Cho (mg/dL)	168.0 ± 17.4	145.5 ± 18.8^‡^	148.8 ± 20.6^‡^	147.5 ± 22.0^‡^	−20.5 ± 18.3
LDL-C (mg/dL)	88.5 ± 12.9	68.0 ± 13.9^‡^	65.3 ± 18.0^‡^	67.9 ± 17.0^‡^	−20.3 ± 15.3
HDL-C (mg/dL)	46.4 ± 11.6	47.8 ± 10.3	51.0 ± 10.3*	51.5 ± 12.1*	4.6 ± 5.9^†^
TG (mg/dL)	165.4 ± 78.9	148.8 ± 78.8	162.3 ± 86.1	140.6 ± 80.7	−21.0 ± 66.1
LDL-C/HDL-C ratio	2.05 ± 0.73	1.51 ± 0.54*	1.33 ± 0.45^‡^	1.39 ± 0.49^‡^	−0.57 ± 0.43
	**Rosuvastatin 2.5 mg/day + ezetimibe 10 mg/day**				
T-Cho (mg/dL)	164.0 ± 23.3	134.7 ± 18.4^‡^	137.5 ± 24.5^‡^	138.5 ± 19.36^‡^	−23.5 ± 17.2
LDL-C (mg/dL)	84.3 ± 14.5	62.3 ± 12.2^‡^	62.6 ± 15.3^‡^	62.9 ± 11.7^‡^	−21.9 ± 14.4
HDL-C (mg/dL)	49.9 ± 12.2	51.0 ± 10.0	51.7 ± 11.0	51.0 ± 9.1	−0.0 ± 6.7
TG (mg/dL)	149.4 ± 103.9	106.7 ± 36.1*	115.7 ± 41.7	123.3 ± 50.0	−7.8 ± 52.8
LDL-C/HDL-C ratio	1.76 ± 0.41	1.26 ± 0.34^‡^	1.22 ± 0.24^‡^	1.27 ± 0.30^‡^	−0.46 ± 0.27

Data are presented as mean ± standard deviation. T-Cho, total cholesterol; TG, triglycerides; LDL-C, low-density lipoprotein cholesterol; HDL-C, high-density lipoprotein cholesterol.

*p < 0.05 versus baseline; ^†^p < 0.05 versus rosuvastatin 2.5 mg/day + ezetimibe 10 mg/day; ^‡^p < 0.0001 versus baseline.

**Figure 2:**
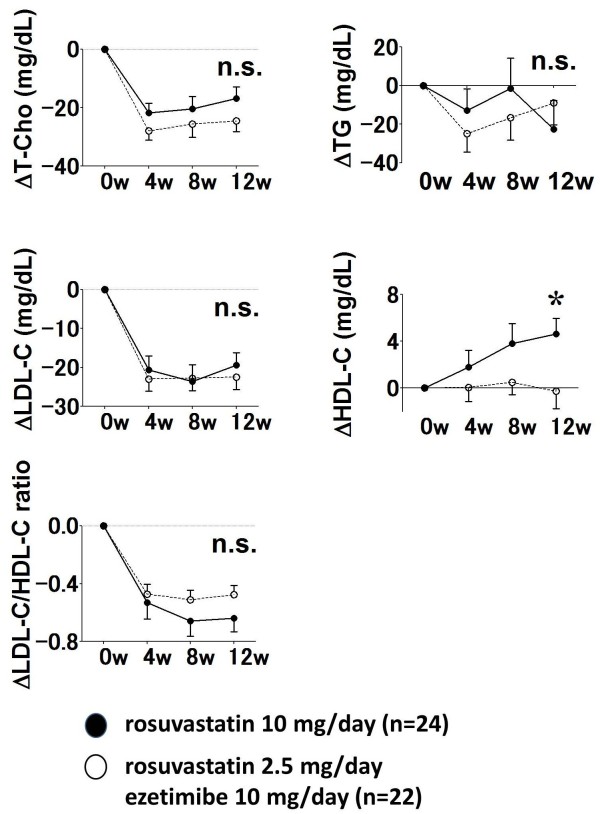
**Changes in lipid profiles between baseline and 4, 8 and 12 weeks after randomization.** HDL-C, high-density lipoprotein cholesterol; LDL-C, low-density lipoprotein cholesterol; T-Cho, total cholesterol; TG, triglycerides. *p < 0.05 versus rosuvastatin 2.5 mg/day + ezetimibe 10 mg/day.

Levels of T-Cho, LDL-C and the LDL-C: HDL-C ratio were significantly decreased in both groups after 4, 8 and 12 weeks (Table 2), but TG did not significantly change in either group. The HDL-C level significantly increased from 46.4 ± 11.6 to 51.5 ± 12.1 mg/dL (p = 0.0105) in the R10 group, but not in the R2.5/E10 group (49.9 ± 12.2 vs. 51.0 ± 9.1 mg/dL, p = 0.9790). Figure 2 shows changes in the lipid profiles between baseline and 4, 8 and 12 weeks after randomization. The treatment strategies similarly reduced LDL-C (Δ LDL-C: -19.4 ± 14.2 vs. -22.4 ± 14.3 mg/dL, p = 0.7049), as well as T-Cho, TG and the LDL-C/HDL-C ratio.

**Figure 3:**
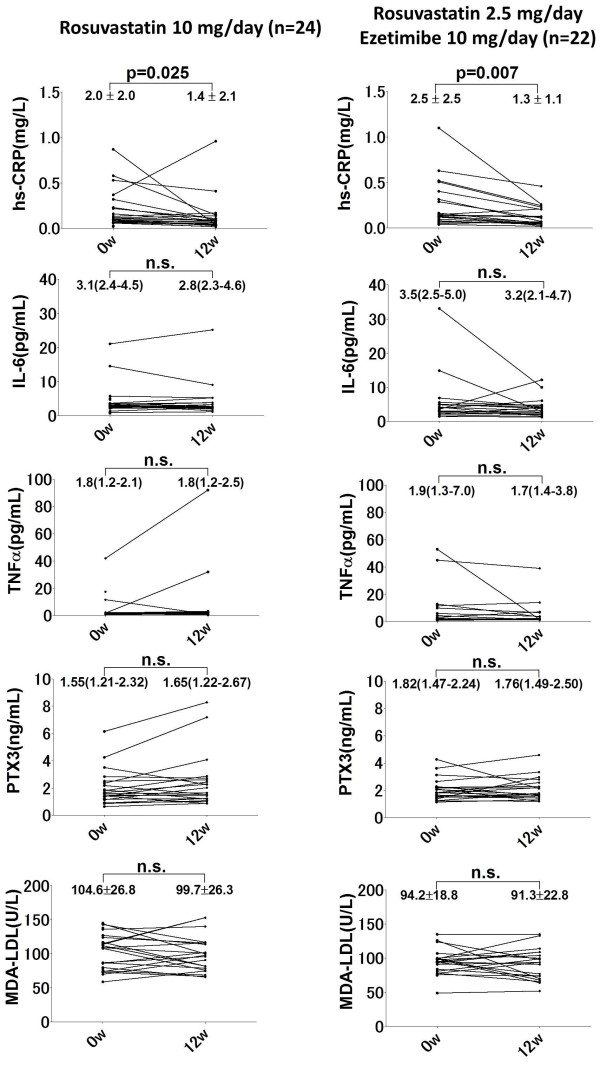
**Changes in inflammatory markers and MDA-LDL levels between baseline and 12 weeks after randomization.** hs-CRP, high sensitivity-C-reactive protein; IL-6, interleukin-6; MDA-LDL, malondialdehyde-modified-low-density lipoprotein; PTX3, pentraxin 3; TNF-α, tumour necrosis factor-α.

Levels of MDA-LDL were similarly reduced in both groups (Figure 3), but R10 increased the HDL-C level more effectively than R2.5/E10 compared with baseline (Δ HDL-C: 4.6 ± 5.9 vs. 0.0 ± 6.7 mg/dL, p = 0.0249).

Among the inflammatory markers, hs-CRP was significantly reduced in both groups at 12 weeks after randomization (R10: 0.20 ± 0.22 vs. 0.08 ± 0.04 mg/dL, p = 0.0167; R2.5/E10: 0.21 ± 0.17 vs. 0.13 ± 0.11 mg/dL, p = 0.0028), whereas IL-6, TNF-α and PTX3 did not significantly change in either group (Table 3).

**Table 3:** Inflammatory markers at baseline and 12 weeks after randomization

	**Rosuvastatin 10 mg/day (n = 24)**	**Rosuvastatin 2.5 mg/day + ezetimibe 10 mg/day (n = 22)**
hs-CRP (mg/L)		
Baseline	2.0 ± 2.0	2.5 ± 2.5
12 weeks	1.4 ± 2.1*	1.3 ± 1.1*
12 weeks - baseline	−1.0 ± 2.5	−1.3 ± 1.9
MDA-LDL (U/L)		
Baseline	104.6 ± 26.8	94.2 ± 18.8
12 weeks	99.7 ± 26.3	91.3 ± 22.8
12 weeks - baseline	−2.7 ± 23.3	−3.9 ± 21.5
IL-6 (pg/mL)		
Baseline	3.1 (2.4 – 4.5)	3.5 (2.5 - 5.0)
12 weeks	2.8 (2.3 – 4.6)	3.2 (2.1 - 4.7)
12 weeks - baseline	0.2 (−0.7 - 0.8)	−0.4 (−1.3 - 0.4)
TNF-α (pg/mL)		
Baseline	1.8 (1.2 - 2.1)	1.9 (1.3 - 7.0)
12 weeks	1.8 (1.2 - 2.5)	1.7 (1.4 - 3.8)
12 weeks - baseline	0.3 (−0.2 - 0.8)	−0.2 (−2.8 - 0.5)
PTX3 (ng/mL)		
Baseline	1.55 (1.21 - 2.32)	1.82 (1.47 - 2.24)
12 weeks	1.65 (1.22 - 2.67)	1.76 (1.49 - 2.50)
12 weeks - baseline	0.12 (−0.09 - 0.98)	0.01 (−0.30 - 0.51)

Data are shows as means ± standard deviation. hs-CRP, high sensitivity-C-reactive protein; IL-6, interleukin-6; MDA-LDL, malondialdehyde-modified-low-density lipoprotein; PTX3, pentraxin 3; TNF-α, tumour necrosis factor-α. *p < 0.05 vs. baseline.

Figure 3 shows levels of inflammatory markers and MDA-LDL at baseline and at 12 weeks after randomization. Levels of hs-CRP were significantly reduced in both groups. However, baseline levels and changes in IL-6 and TNF-α varied widely compared with hs-CRP. Changes in hs-CRP levels after treatment did not significantly differ between the two groups (−0.8 ± 2.5 vs. -1.3 ± 1.9 mg/L, p = 0.4297). No other inflammatory markers or MDA-LDL significantly differed between the two groups.

## Discussion

This is the first randomized controlled comparison of inflammatory markers between therapy with quadruple-dose rosuvastatin and rosuvastatin combined with ezetimibe when both methods similarly reduced LDL-C. We assumed based on previous findings [18-20] that both R10 and R2.5/E10 would equally reduce LDL-C. We found that both treatments indeed similarly reduced LDL-C as well as T-Cho, TG, LDL-C/HDL-C ratio and MDA-LDL after 12 weeks. However, HDL-C was significantly improved in the R10, compared with the R2.5/E10 group. Changes in inflammatory markers between the two groups did not significantly differ. Only HDL-C among all tested parameters significantly differed between the two groups. We included patients who had hs-CRP > 1.0 mg/L despite treatment with rosuvastatin 2.5 mg. The cut-off value was defined as hs-CRP > 1.0 mg/L based on the population-based, prospective, cohort Hisayama study [17], which demonstrated that hs-CRP > 1.0 mg/L is the cut-off for a high risk of future CAD development in the general Japanese population. This value is much lower than the hs-CRP value of >3.0 mg/L that corresponds to a high risk for future cardiovascular events in non-Japanese populations [23]. None of the participants in both of these studies were taking statins. The PATROL trial compared the safety and efficacy of atorvastatin, rosuvastatin and pitavastatin head-to-head in patients with hypercholesterolemia [24] and found that hs-CRP after treatment with rosuvastatin 2.5 mg was 1.1 ± 2.0 mg/L in Japanese patients with CAD. This suggested that very few of our patients had hs-CRP > 3.0 mg/L after treatment with rosuvastatin 2.5 mg. Therefore, our cut-off of hs-CRP > 1.0 mg/L was reasonable for our participants.

A simvastatin study with a similar protocol to the present study found no significant improvements in hs-CRP and IL-6 [25]. Likewise, changes in inflammatory markers did not significantly differ between R10 and the R2.5/E10 in the present study, in which the protocol was designed so that both strategies would similarly reduce LDL-C. The findings of the present and simvastatin studies suggest that the anti-inflammatory effects and the LDL-C reductions do not significantly differ between a quadruple dose of any statin and the addition of ezetimibe.

We found that both strategies significantly reduced hs-CRP, although pro-inflammatory cytokines did not significantly differ between baseline and 12 weeks later. Some small-scale studies have found that statins significantly reduce IL-6 and TNF-α [26,27]. On the other hand, the population-based Colaus study that examined associations between statins and hs-CRP, IL-6 and TNF-α in 6,184 patients, found lower hs-CRP levels in those treated with, than without statins, and that statins did not elicit any effects on IL-6 and TNF-α levels in the patients [28]. The present and Colaus studies showed that statins decrease hs-CRP without affecting pro-inflammatory cytokines. The reason for the discrepancy between hs-CRP and pro-inflammatory cytokines remains undetermined. More recently, another study found that neither high-dose simvastatin nor low-dose simvastatin combined with ezetimibe reduce proinflammatory markers such as IL-6 [29]. In addition, several studies in vitro have demonstrated a direct effect of statins on IL-6 induced hs-CRP expression in human hepatocytes [30-32]. Although the anti-inflammatory effect of ezetimibe monotherapy has been controversial [33], our results suggest that R10 and R2.5/E10 can decrease hs-CRP through mechanisms that are independent of the IL-6 receptor.

The present study showed that the novel inflammatory marker PTX3, which is a member of pentraxin superfamily like hs-CRP, did not significantly differ between plasma levels at baseline and at 12 weeks after randomization. By contrast, other studies have shown that statin therapy significantly decreases PTX3 levels [34,35]. One possible explanation for the discrepancy is that the baseline PTX3 concentration was similar to the average in healthy volunteers [36]. The plasma PTX3 concentration might have been fully decreased in our patients at the time of enrolment in the present study, because they had already been treated with rosuvastatin (2.5 mg/day) for at least 4 weeks before randomization.

The results of the present study were similar to those of a comparison of atorvastatin (10 mg/day) with atorvastatin (10 mg/day) combined with ezetimibe (10 mg/day) in patients with CAD in that the combination significantly decreased hs-CRP, but not PTX3 [37]. Therefore, adding ezetimibe might not decrease PTX3 in the manner of hs-CRP.

The present study found that R10 significantly increased HDL-C by 6.9% (1.8% - 15.1%) from baseline compared with R2.5/E10. Our results were similar to those of a meta-analysis in the VOYAGER study, which showed that rosuvastatin (10 mg/day) increases HDL-C by 6.1 ± 0.5% from baseline [38]. In contrast, others have found that adding ezetimibe does not significantly increase HDL-C from baseline [25,39,40]. Therefore, these results suggest that increasing the dose of statin elevates HDL-C more effectively than adding ezetimibe. The TNT trial of individuals in whom atorvastatin decreased LDL-C to <70 mg/dL [41] showed that low HDL-C levels remain as independent predictors of CAD risk even in patients with low LDL-C. Both R10 and R2.5/E10 decreased LDL-C to <70 mg/dL in the present study, as in the TNT trial, and the only significant difference was HDL-C between the two groups. The clinical benefit of increasing HDL-C using antidyslipidemic agents was controversial. According to the recent trial [42], cholesteryl ester transfer protein inhibitor which increased HDL-C levels 31 to 40% did not improve clinical outcome in patients with CAD. However, another trial [43] of the clinical value of increasing HDL-C using statin found that a change in the HDL-C level was a powerful independent risk factor for cardiovascular events. Although the clinical value of increasing HDL-C was undetermined, these trials suggested that statins had characteristic effects to improve both HDL-C levels and clinical outcomes. Thus, our results might reflect the difference in clinical outcomes between high-dose rosuvastatin monotherapy and the combination of ezetimibe and rosuvastatin.

The lack of additional benefits of ezetimibe beyond LDL-C and hs-CRP lowering might partly explain the findings of ENHANCE study [44] in which ezetimibe, when added to a statin, did not alter the progression of carotid artery intima-media thickening despite a further reductions in LDL-C and in inflammatory biomarkers such as hs-CRP compared with statins alone. Although the patients enrolled in the ENHANCE study had relatively low levels of carotid artery intima-media thickening and a correlation between carotid artery intima-media thickening change and cardiovascular outcome was not investigated, adding ezetimibe to statin did not reduce carotid artery intima-media thickness. On the other hand, others have shown that statins cause carotid intima-media thickness to regress [45,46]. According to these and the present findings, quadruple-dose statin might be more favourable than a combination of ezetimibe and statin at least from the viewpoint of an anti-atherogenic effect.

### Study limitations

The present study has several limitations. Firstly, statistical power was low because we sampled only 46 patients, of whom only a few had hs-CRP > 1.0 mg/L while taking 2.5 mg/day of rosuvastatin. However, the findings of this exploratory pilot study were meaningful for planning a future large-scale study. Secondly, since all the patients were Japanese and because the cut-off for a high risk of CAD development is lower for Japanese than for other patients, ethnic variation should be taken into account when considering changes in inflammatory markers. Thirdly, this open label, but not double-blind, study examined surrogate endpoints of lipid profiles and inflammatory markers and did not measure any actual clinical outcomes. Further prospective long-term large clinical trials are needed to define the effects of statins and ezetimibe on clinical outcomes.

## Conclusions

Inflammatory markers did not significantly differ in patients with CAD taking rosuvastatin 2.5 mg regardless of whether they were changed to a quadruple dose or a combination of rosuvastatin and ezetimibe. However, quadruple-dose rosuvastatin alone can elevate HDL-C more effectively than rosuvastatin combined with ezetimibe under conditions of an equal reduction in LDL-C.

## Abbreviations

CAD: Coronary artery disease; HDL-C: High-density lipoprotein cholesterol; hs-CRP: High sensitive C-reactive protein; IL-6: Interleukin-6; LDL-C: Low-density lipoprotein cholesterol; MDA-LDL: Malondialdehyde-modified low-density lipoprotein cholesterol; PTX3: Pentraxin 3; R2.5/E10: Rosuvastatin (2.5 mg/day) plus ezetimibe (10 mg/day); R10: Rosuvastatin 10 mg/day; T-Cho: Total cholesterol; TG: Triglycerides; TNF-α: Tumour necrosis factor-α.

## Competing interests

There are no conflicts of interest or competing interests associated with this study.

## Authors’ contributions

DY and MI conceived the study, participated in the study design, statistical analysis, data interpretation and in writing the manuscript. The first two authors contributed equally to this work. KN conceived the study, participated in study design and data collection. YO, YT, TaK, KI and ToK participated in the study design and data collection. HW and HI participated in study design, statistical analysis, data interpretation and in writing the manuscript. All authors have read and approved the final manuscript.
